# Insights into *Aspergillus fumigatus* morphogenesis and pathogenesis through the putative lipid transporter ArvA

**DOI:** 10.1128/msphere.00853-25

**Published:** 2026-05-15

**Authors:** Cecilia Gutierrez-Perez, Jane T. Jones, Charles T. S. Puerner, Sandeep Vellanki, Nicole E. Kordana, Matthew R. James, Elisa M. Vesely, Angus Johnson, Robert A. Cramer

**Affiliations:** 1 Department of Microbiology and Immunology, Geisel School of Medicine at Dartmouth, Hanover, New Hampshire, USA; University of Guelph, Guelph, Ontario, Canada

**Keywords:** *Aspergillus fumigatus*, lipids, antifungal susceptibility, fungal pathogenesis

## Abstract

*Aspergillus fumigatus* poses a significant threat to human well-being, in part due to the increasing emergence of strains resistant to frontline antifungal therapy. In this study, we observed that the gene *arvA* is required for *A. fumigatus* morphogenesis, antifungal drug susceptibility, and cell wall homeostasis. Intriguingly, our study reveals novel morphological and growth aberrations in the absence of *arvA*. Loss of *arvA* results in hyper-swollen conidia that give rise to stunted, polarity-deficient hyphae in numerous environmental conditions, indicating a pivotal role for *arvA* in *A. fumigatus* morphogenesis. Surprisingly, despite these severe *in vitro* morphological and cell wall defects, *arvA* was not required for morbidity and mortality in immunologically distinct murine models of invasive pulmonary aspergillosis. However, the mechanisms by which the *arvA* mutant can cause disease differ between the two models. Growth in natural calf lung surfactant was able to somewhat normalize ∆*arvA* growth with the wild-type strain, suggesting lung surfactant may partially complement the severe *in vitro* morphological defects of *arvA* loss *in vivo*. Taken together, our observations reveal *arvA* as a mediator of *A. fumigatus* antifungal drug susceptibility and highlight the complex and ill-defined pulmonary nutrient environment’s role in mediating *A. fumigatus* pathogenesis and disease progression.

**IMPORTANCE:**

*Aspergillus fumigatus* is a challenging fungal pathogen in the clinic, in part due to increasing azole drug resistance. In this study, we observed that the loss of the *A. fumigatus* gene *arvA* results in increased azole susceptibility and significant *in vitro* morphological changes highlighted by hyper-swollen conidia that yield stunted and polarity-deficient hyphae. Importantly, despite these severe *in vitro* morphological and growth abnormalities, ∆*arvA* surprisingly retains full pathogenicity and virulence in two immunologically distinct murine models of invasive pulmonary aspergillosis. These results challenge our understanding of the in-host environment and how it mediates fungal morphogenesis and pathogenesis. These results, consequently, not only enhance our understanding of the role of *arvA* in *A. fumigatus* morphogenesis and drug susceptibility but also further emphasize the importance of *in vivo* animal models in fully evaluating potential antifungal drug targets.

## INTRODUCTION

*Aspergillus fumigatus* is a filamentous fungus that can be a pathogen in many hosts with compromised or altered immune system function. In 2024, *A. fumigatus* was estimated to cause upwards of 2 million cases of invasive aspergillosis per year worldwide, with a crude mortality of ~85% (1). The clinical presentation of invasive aspergillosis is characterized by extensive hyphal growth that often results in the formation of biofilms in tissue. Hyphae and associated biofilms present a unique challenge to not only the immune system but also contemporary antifungal therapy treatments (2). Consequently, studies of hyphal morphogenesis and biofilm development are important to identify mechanisms amenable to *in vivo*-relevant antifungal therapeutic development.

The endoplasmic reticulum (ER)-resident protein Arv1 functions in ceramide, sterol, and GPI-anchor precursor transport, sphingolipid metabolism, ER integrity, fatty acid resistance, hyphal formation, virulence, autophagy, and cell cycle progression, all processes critical for biofilm development (3–11). The *ARV1* gene is conserved among eukaryotic species, with its physiological function as a putative lipid transporter conserved between pathogenic and non-pathogenic fungi (12). Arv1 has been extensively characterized in *Saccharomyces cerevisiae*, *Candida albicans,* and in the context of human disease (3–6, 9–20), but its role in *A. fumigatus* hyphal morphogenesis and pathogenicity is not defined.

In *C. albicans,* Arv1p is necessary for proper sterol distribution, hyphal growth, virulence, and azole sensitivity (5, 6). Consistent with *S. cerevisiae* synthetic lethal analyses, loss of *C. albicans ARV1* results in azole hypersusceptibility. Increased azole susceptibility in the absence of *ARV1* is likely due to Arv1p forming a heterodimer with Erg11p, the target for azole drugs, to stabilize Erg11p and increase its half-life (6, 21). Arv1 mutants that were unable to interact with Erg11p were avirulent and hypersusceptible to azoles, suggesting that this interaction is necessary for causing disease *in vivo* and azole susceptibility (6). Loss of Arv1p also results in Erg11p mislocalization (6). In *S. cerevisiae*, loss of *ARV1* results in defective ceramide transport from the ER to the Golgi, which infringes on sphingolipid metabolism (3, 4). Furthermore, synthesis of GPI anchors is compromised in ∆*arv1* mutants (3). Arv1p interacts with GPI biosynthetic machinery and has been postulated to be a GPI flippase (13, 22). Δ*arv1* has similar phenotypes to GPI anchor synthesis mutants, such as increased accumulation of chitin, hypersusceptibility to the chitin inhibitor calcofluor white (CFW), and accumulation of GPI-anchor precursors (3, 23). In *S. cerevisiae,* Δ*arv1* cells over-accumulate sterols in the ER while lacking sterols in the plasma membrane (4, 11). As a result, ER integrity and morphology are compromised in ∆*arv1,* leading to increased UPR activation (10). This result is consistent with Arv1p’s hypothesized role as a retrograde sterol transporter.

Arv1’s roles in lipid homeostasis and GPI anchor synthesis are conserved in human cells (11, 15, 19, 20). Outside the roles reported in fungi, Arv1 contributes to cell cycle progression in human cells (8). Arv1 can recruit multiple proteins necessary for cell cycle progression to the cleavage furrow, including myosin (8). Lack of Arv1 resulted in delayed telophase progression and an increase in multinucleate cells (8). These phenotypes are ill-defined in fungi but are suggested to be independent of Arv1’s roles in lipid homeostasis (8).

In this work, we identified the *Aspergillus fumigatus* Arv1 homolog, herein named *arvA*. We hypothesized that loss of *arvA* would impact hyphal morphogenesis, biofilm formation, antifungal drug susceptibility, and virulence in a mouse model of invasive aspergillosis. Loss of *arvA* in *A. fumigatus* generated phenotypes similar to those observed in other fungal species upon Arv1p loss, such as azole susceptibility, delay in hyphal formation, changes in cell wall composition, and an inability to grow at high temperatures. However, loss of *arvA* in *A. fumigatus* revealed distinct and previously unreported morphological changes in agar-based and submerged biofilms *in vitro*. Strains lacking *arvA* are unable to form hyphae that grow radially in agar-based colony biofilms and instead grow as compact micro-colonies. In a submerged biofilm model, ∆*arvA* conidia are hyper-swollen and multinucleate, with a loss of polarity and conidia reaching a size 10–12 times larger than wild-type and reconstituted strains. Surprisingly, in spite of ∆*arvA’s* striking stunted morphology *in vitro*, ∆*arvA* is able to grow *in vivo* in the mouse lung and has full virulence as measured by murine mortality in two immunologically distinct invasive pulmonary aspergillosis (IPA) murine models. We observe that *in vitro* growth in natural calf lung surfactant is able to partially complement the morphology and growth defects associated with loss of *arvA*, which suggests that the carbon/nutritional environment in the lung normalizes growth and virulence in the absence of *arvA*. These results highlight the gap between *in vitro* laboratory conditions and the physiological conditions *in host*.

## RESULTS

### Loss of function of *arvA* (AFUB_027230) results in striking morphological changes

We became interested in the *A. fumigatus ARV1* homolog, AFUB_027230 (herein called *arvA,* consistent with *A. fumigatus* gene nomenclature convention), due to its synthetic lethal interaction with *ERG11* in *S. cerevisiae* (24). Loss of function of *S. cerevisiae* Arv1p increases susceptibility to alkaline pH, azoles, statins, allylamines, and calcofluor white (6, 20, 25–27). ArvA is a likely ortholog to human Arv1 (BLASTp 23% query cover with 33.02% identity) and *S. cerevisiae* Arv1p (BLASTp 16% query cover with 50.7% identity) as indicated by reciprocal BLAST queries. ArvA also has the predicted canonical ARV1 domain. Subsequently, we generated an *arvA* null mutant strain (∆*arvA*) and a reconstituted strain (∆*arvA + arvA*) using laboratory strain CEA10 as the parental strain and a CRISPR/Cas9-mediated approach (primers indicated in Table S1).

**Fig 1:**
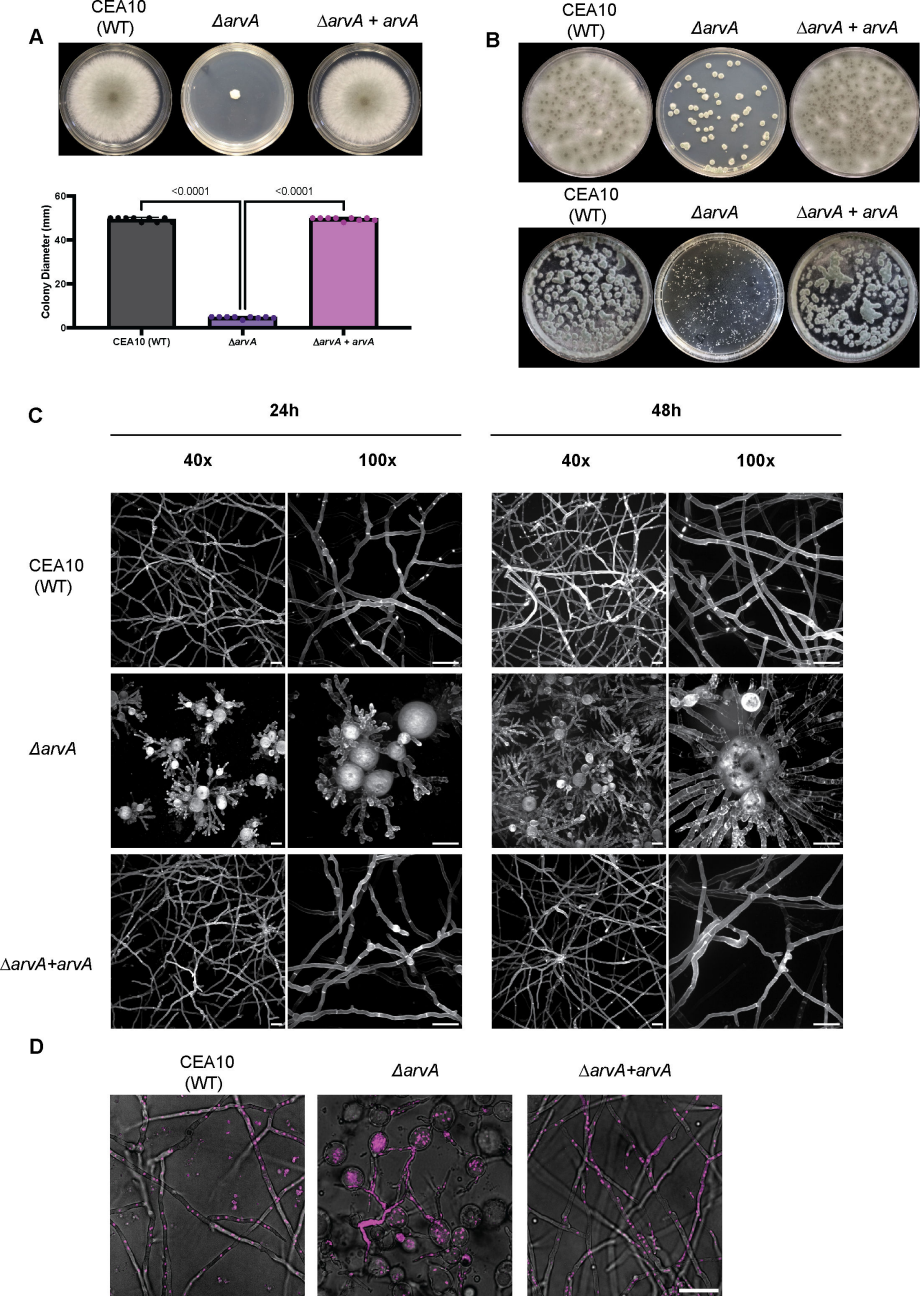
Loss of *Aspergillus fumigatus arvA* induces morphological changes on agar and in submerged colony biofilms. (**A**) ∆*arvA* has significantly decreased growth on solid medium compared to the wild-type (CEA10) and the reconstituted strains. A total of 1 × 10^3^ conidia in 2 µL of CEA10, ∆*arvA,* and ∆*arvA + arvA* strains were inoculated onto GMM plates and incubated for 72 hours at 37°C with 5% CO_2_. Colony diameter was measured using a ruler and reported as millimeters. Images and data are representative of three biological replicates with three technical replicates each. Statistical significance was determined using an ordinary one-way ANOVA with Tukey’s multiple comparisons test. (**B**) ∆*arvA* grows as micro-aggregates in solid and liquid media. The ∆*arvA* strain was inoculated onto solid (top panel, 50 μL of a 1 × 10^3^ conidia/mL stock) or liquid (bottom panel, 1 × 10^5^ conidia/mL) GMM and incubated at 37°C with 5% CO_2_ for 72 hours for solid medium and 48 hours for liquid medium. The images are representative of multiple independent experiments. (**C**) ∆*arvA* has hyper-swollen conidia, loss of polarity, decreased hyphal growth, and multiple germ tube emergence in submerged biofilms compared to wild-type and reconstituted strain. A total of 1 × 10^5^ conidia/mL of CEA10, ∆*arvA,* and ∆*arvA + arvA* were inoculated in filtered liquid GMM and incubated at 37°C with 5% CO_2_ for 24 and 48 hours. Biofilms were stained with 25 µg/mL of calcofluor white approximately 20 minutes prior to imaging. Images were taken on a Nikon spinning disk confocal microscope at 40× and 100× magnification, using a 405 nm laser to visualize the CFW signal. Z-stacks were taken that encompassed the growth of the *arvA* null mutant, and max projections of those Z-stacks are shown. Scale bars are 50 µm. Raw images were processed in FIJI as indicated in Materials and Methods. (**D**) ∆*arvA* hyper-swollen conidia have multiple Hoescht-stained puncta. CEA10, ∆*arvA,* and ∆*arvA + arvA* were inoculated in filtered liquid GMM and incubated at 37°C with 5% CO_2_ for 24 hours. Biofilms were fixed with paraformaldehyde and permeabilized with Triton before being stained with Hoescht for 15 minutes prior to imaging. Images were taken on a Nikon Ti-E-inverted spinning disk microscope at 40× magnification, using a 405 nm laser to visualize the Hoescht signal. Z-stacks were taken that encompassed the growth of the *arvA* null mutant, and max projections of those Z-stacks are shown. Scale bar = 25 µm. Raw images were processed in FIJI as indicated in Materials and Methods.

Following confirmation of gene replacement and reconstitution, isogenic strain colony biofilm growth was observed on glucose minimal medium (GMM) petri plates incubated for 72 hours at 37°C with 5% CO_2_ (Fig. 1A). After 72 hours, ∆*arvA* exhibited a significant decrease in hyphal growth as evidenced by a dramatic decrease in colony diameter compared to strains CEA10 and ∆*arvA + arvA* (Fig. 1A). The parental (CEA10) and reconstituted strain (∆*arvA + arvA*) grow to about 50 mm colony diameter after 72 hours, while ∆*arvA* only reaches 4.8 mm (Fig. 1A). This result suggests that ArvA is necessary for normal colony biofilm development in commonly used laboratory *A. fumigatus* culture conditions. Additionally, when conidia from CEA10 and ∆*arvA + arvA* are plated on solid medium, hyphae grow closely together and are indistinguishable from each other as a lawn (Fig. 1B). Interestingly, the ∆*arvA* strain colonies grow as dense micro-colony aggregates and do not form a colony biofilm (Fig. 1B). Dense aggregate formation is also observed at the air-liquid interface of submerged ∆*arvA* biofilms grown in liquid GMM (Fig. 1B).

After observing the striking colony growth differences between ∆*arvA* and the parental and reconstituted strains, we assessed strain growth and morphology in a submerged biofilm model. *A. fumigatus* dormant conidia size is approximately 2 µm, and upon the start of germination, conidia isotropically swell to ~5 µm (28). After swelling, one germ tube develops from which hyphal growth begins. A second germ tube develops on the conidia on the opposite side of the first one, and continued hyphal growth develops a biofilm on a surface. At 24 hours, CEA10 and ∆*arvA + arvA* exhibited normal biofilm development at 40× magnification, and there were only two germ tubes per spore when assessing morphology at 100× magnification (Fig. 1C). Loss of *arvA (*∆*arvA*) drastically alters submerged biofilm morphology. When compared to the parental and reconstituted strains, ∆*arvA* 24 hour biofilms have significantly decreased hyphal length (Fig. 1C). Most striking, conidia of the ∆*arvA* strain are hyper-swollen, and a closer examination of the conidia at 100× magnification revealed multiple germ tubes emerging per conidia with hyphal hyperbranching, indicating a loss of polarity (Fig. 1C). At 48 hours, the ∆*arvA* strain continued to exhibit hyper-swollen conidia, a multitude of germ tubes per conidia, and hyphal hyperbranching that were not observed in CEA10 and ∆*arvA + arvA* (Fig. 1C). Combined, these results suggest that ArvA is necessary for both polarity establishment and maintenance in *A. fumigatus* and consequently robust biofilm formation.

These drastic changes in morphology observed in *A. fumigatus* ∆*arvA* have not been reported in yeast; therefore, the existing fungal literature on Arv1 failed to provide a clear explanation for ∆*arvA* hyper-swollen conidia phenotype in submerged biofilms. However, human Arv1 has been observed to play a role in cell cycle telophase progression, and the lack of Arv1 in human cells results in multinucleate cells (8). An accumulation of nuclei as a result of cell cycle progression perturbation could explain the size increase observed in ∆*arvA* conidia. We therefore hypothesized that the large size of the hyper-swollen ∆*arvA* conidia is in part due to an expansion in the number of nuclei. To test this hypothesis, 24 hour biofilms of the isogenic strain set were exposed to Hoescht. In support of the hypothesis, ∆*arvA* hyper-swollen conidia contained multiple distinct Hoescht-stained puncta suggestive of increased nuclei within each hyper-swollen conidia (Fig. 1D). From these data, we conclude that *A. fumigatus* ArvA is critical for normal cell cycle progression and conidia germination under the conditions examined.

### ∆*arvA* displays susceptibility to cell wall and cell membrane stressors

**Fig 2:**
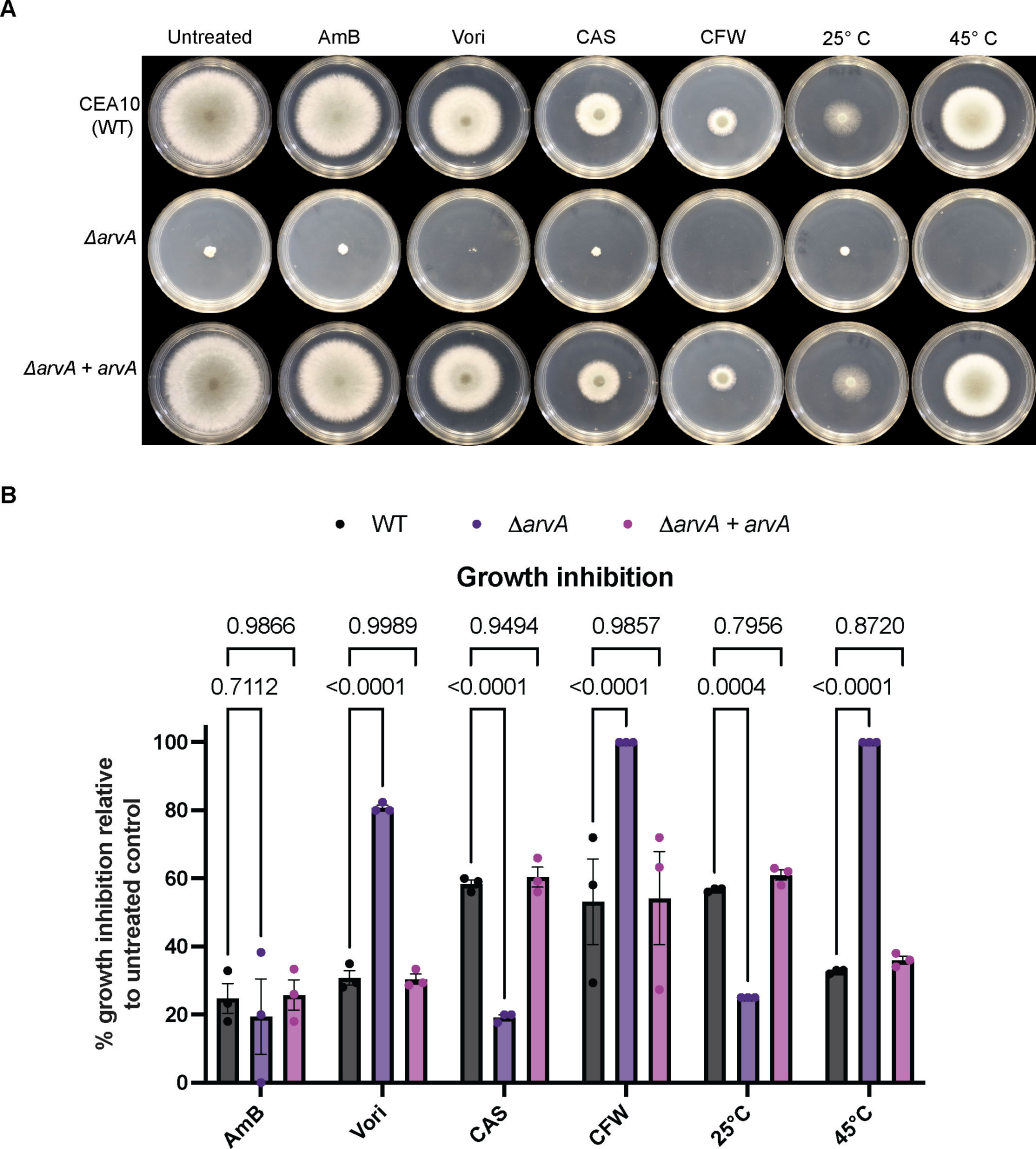
Loss of *A. fumigatus arvA* alters susceptibility to cell membrane and cell wall-targeting antifungal agents. (**A and B**) ∆*arvA* is more susceptible to high temperature, voriconazole, and calcofluor white and less to low temperature and caspofungin, relative to wild-type and reconstituted strains in agar-based colony assays. A total of 1 × 10^3^ conidia in 2 µL of CEA10, ∆*arvA,* and ∆*arvA + arvA* strains were inoculated onto GMM plates treated with amphotericin B (AmB; 0.75 µg/mL), voriconazole (Vori; 0.0625 µg/mL), caspofungin (CAS; 0.125 µg/mL), or calcofluor white (CFW; 25 µg/mL) and incubated for 72 hours at 37°C with 5% CO_2_. In parallel, all three strains were inoculated onto GMM agar plates and grown under the same conditions except at either 25°C or 45°C. Colony diameter was measured using a ruler and normalized to the percentage of growth inhibition relative to its untreated control. Images and data are representative of three biological replicates with three technical replicates each. Statistical significance was determined using an ordinary two-way ANOVA with Dunnett’s multiple comparisons test.

After establishing that ∆*arvA* in *A. fumigatus* is an important determinant of polar growth and morphology, we next tested the impacts of these defects on pathogenicity and antifungal-related phenotypes. One pathogenicity-related phenotype attributed to the loss of Arv1 is a loss of thermotolerance (11, 29, 30). To test whether *A. fumigatus* ∆*arvA* exhibited an inability to grow at elevated temperatures, we employed an agar-based colony biofilm assay (Fig. 2A). We inoculated 1 × 10^3^ conidia of CEA10, ∆*arvA,* and ∆*arvA + arvA* onto GMM plates and incubated for 72 hours at 25°C, 37°C, or 45°C with 5% CO_2_. CEA10 and ∆*arvA + arvA* strains were able to grow at each respective temperature with no growth or morphological differences (Fig. 2A). While ∆*arvA* growth at 25°C did not look markedly different compared to 37°C, the mutant was strikingly not able to grow at 45°C (Fig. 2A). This result confirms a role for *A. fumigatus* ArvA-mediated growth at temperatures found above human body temperatures and that growth and morphology aberrations exist in the absence of ArvA at both ambient and mammalian core body temperatures.

As growth at high temperatures requires alterations to membrane sterol and lipid composition, morphological and growth aberrations associated with loss of ArvA could be due to its predicted role as a lipid transporter. As ArvA has previously been shown to play a role in sterol and sphingolipid metabolism, we hypothesized that loss of *arvA* would alter susceptibility to membrane-targeting antifungals. Due to the striking morphological differences in modes of growth exhibited by ∆*arvA* relative to its parental (CEA10) and reconstituted strain (∆*arvA + arvA),* we employed two different assays to assess susceptibility to cell wall and cell membrane stressors. Furthermore, as the cell membrane directly impacts cell wall homeostasis, we hypothesized that loss of *arvA* would also result in altered susceptibility to cell wall-perturbing agents. We first used an agar-based colony biofilm assay to assess susceptibility to amphotericin B (cell membrane stressor), voriconazole (cell membrane stressor), calcofluor white (cell wall stressor), and caspofungin (cell wall stressor). We inoculated 1 × 10^3^ conidia of CEA10, ∆*arvA,* and ∆*arvA + arvA* onto GMM plates treated with either amphotericin B (0.75 µg/mL), voriconazole (0.0625 µg/mL), caspofungin (0.125 µg/mL), or calcofluor white (25 µg/mL) and incubated for 72 hours at 37°C with 5% CO_2_ (Fig. 2A). Radial growth of each plate was measured and normalized to the percentage of their own untreated control. Treatment with amphotericin B reduced growth by ~25% in both CEA10 and ∆*arvA + arvA* strains and by ~19% in ∆*arvA,* with no statistical difference between the three strains observed (Fig. 2A and B). In contrast, a sub-MIC concentration of voriconazole (0.0625 µg/mL = 1/4–1/8 MIC in wild-type parental strain CEA10) reduced growth about 30% in CEA10 and ∆*arvA + arvA,* compared to ~81% reduction in growth exhibited by ∆*arvA* (Fig. 2A and B). This increase in susceptibility to azoles in the absence of ArvA orthologs has been reported in *S. cerevisiae* and *Candida albicans* (6, 12, 26).

We next tested whether ∆*arvA* had altered susceptibility to the cell wall stress agents calcofluor white (CFW), a chitin-binding molecule, and caspofungin, a β-glucan synthase inhibitor. Caspofungin inhibited CEA10 and ∆*arvA + arvA* growth ~58% and ~60%, respectively, compared to only ~19% growth inhibition of ∆*arvA* (Fig. 2A and B). The ∆*arvA* strain was therefore less susceptible to caspofungin-mediated β-glucan synthesis inhibition. This result suggests that ∆*arvA* has changes in cell wall composition and/or the stress response to echinocandin treatment. In support of the ∆*arvA* strain having a different cell wall composition in comparison to parental and reconstituted strain, ∆*arvA* was hypersusceptible to the chitin-binding agent calcofluor white (Fig. 2A and B). CFW inhibited the growth of CEA10 and ∆*arvA + arvA* strains by ~53%–54%, respectively, compared to a striking 100% growth inhibition of ∆*arvA* (Fig. 2A and B). Overall, ∆*arvA* has altered susceptibility to voriconazole, caspofungin, and CFW compared to its parental and reconstituted strain. However, these assays are potentially limited by the small radial growth exhibited by the ∆*arvA* strain (~5 mm) on solid agar; therefore, any small change in radial growth when treated with a drug is amplified when normalized to the untreated strain.

**Fig 3:**
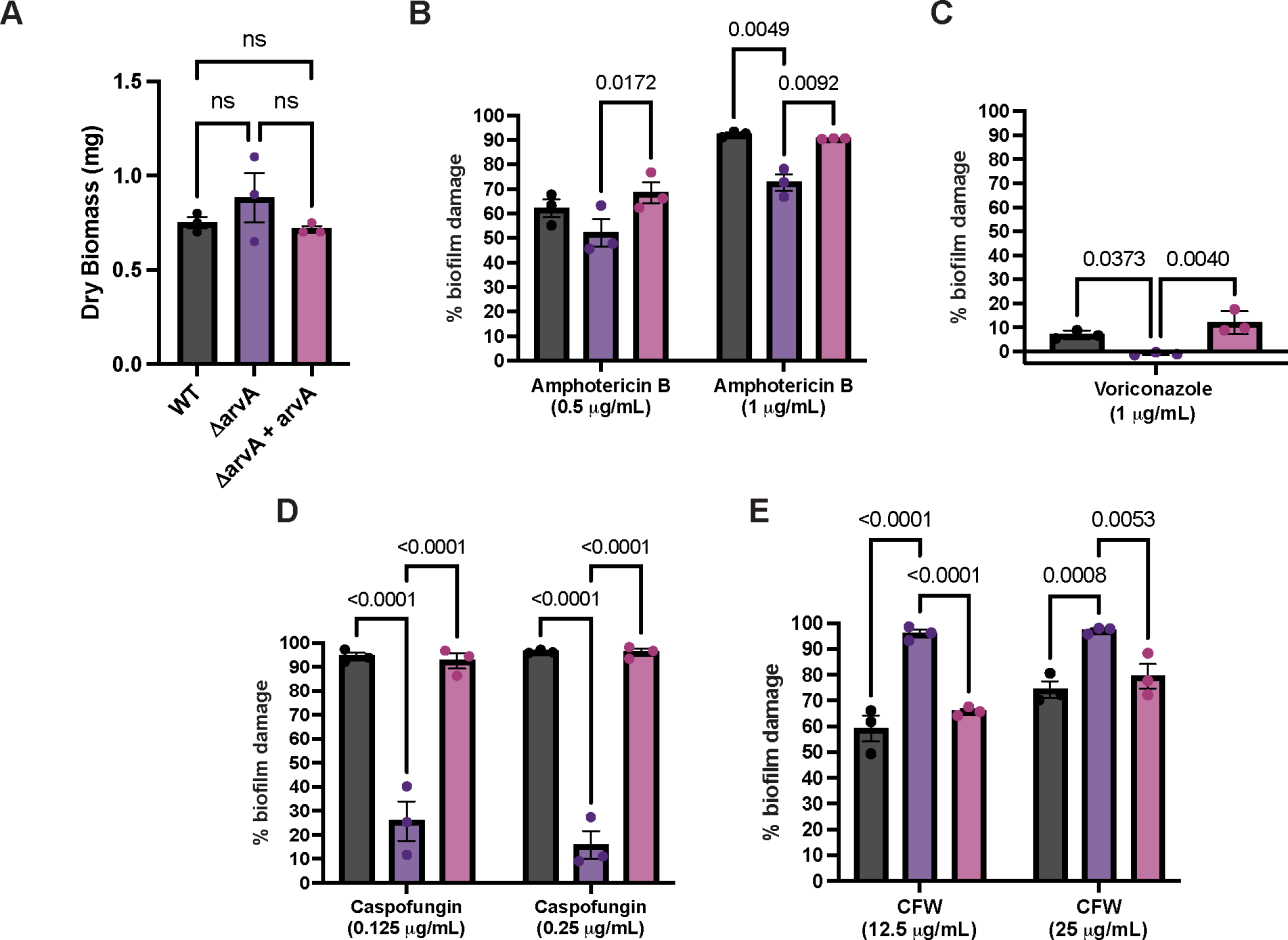
Loss of *A. fumigatus arvA* alters susceptibility to cell membrane and cell wall-targeting antifungal agents in submerged biofilms. (**A**) ∆*arvA* 34 hour biofilm biomass is equivalent to a 16 hour CEA10 and ∆*arvA + arvA* biofilm. A total of 1 × 10^5^ conidia/mL of each strain was inoculated onto 6-well plates and incubated for 34 hours (∆*arvA*) or 16 hours (CEA10, ∆*arvA + arvA*) at 37°C with 5% CO_2_. Biofilm biomass was determined by collecting biomass from 6-well plates into a tube, washing two times with water, lyophilizing, and weighing. Data are representative of three biological replicates, with two to three technical replications each. One-way ANOVA with multiple comparisons. (**B–E**) ∆*arvA* is more susceptible to amphotericin B, voriconazole and calcofluor white, and is less to caspofungin relative to wild-type and reconstituted strains in submerged biofilms as measured by an XTT assay. A total of 1 × 10^5^ conidia/mL of CEA10, ∆*arvA,* and ∆*arvA + arvA* strains were inoculated into liquid GMM and incubated for 34 hours (∆*arvA*) or 16 hours (CEA10, ∆*arvA + arvA*) at 37°C with 5% CO_2_. Medium was removed, and biofilms were treated with voriconazole (0.0625 µg/mL), amphotericin B (0.75 µg/mL), calcofluor white (25 µg/mL), caspofungin (0.125 µg/mL), or vehicle control in liquid GMM for 3 hours. After treatment, the medium was removed, and metabolic activity was determined using XTT as described in Materials and Methods. To assess the effect of drug treatment, metabolic activity was normalized to percent reduction in metabolic activity relative to untreated control (biofilm damage). Data are representative of the mean of each technical replicate from three biological replicates. Statistical significance was determined using two-way ANOVA with Tukey’s multiple comparisons test.

Consequently, to determine if these findings are generalizable to another fungal growth model, we tested the impact of these stress agents using the *A. fumigatus* submerged biofilm model and a 2,3-bis-(2-methoxy-4-nitro-5-sulfophenyl)-2H-tetrazolium-5-carboxanilide (XTT)-based cell damage assay. As XTT readouts may be proportional to total fungal biomass, we first determined that a 34 hour ∆*arvA* biofilm had equivalent biomass to 16 hour CEA10 and ∆*arvA + arvA* biofilms and utilized these time points in subsequent assays (Fig. 3A). We grew ∆*arvA* (34 hours), CEA10 (16 hours), and ∆*arvA + arvA* (16 hours) biofilms in liquid GMM prior to treatment with either amphotericin B (0.5, 1 µg/mL), voriconazole (1 µg/mL), caspofungin (0.125, 0.25 µg/mL), or CFW (12.5, 25 µg/mL) for 3 hours. To assess the effect of drug treatment, metabolic activity (XTT reduction) was normalized to percent reduction in metabolic activity relative to untreated control (biofilm damage) (2). Treatment with 0.5 µg/mL amphotericin B led to a ~62% and ~68% reduction of metabolic activity in CEA10 and ∆*arvA + arvA* biofilms, respectively, compared to a ~52% reduction in ∆*arvA* (Fig. 3B). This modest decrease in metabolic activity reduction observed in the ∆*arvA* strain was aggravated and became statistically significant with an increased concentration of amphotericin B (1 µg/mL) (Fig. 3B). Treatment with 1 µg/mL of amphotericin B reduced metabolic activity by ~90% and ~92% in CEA10 and ∆*arvA + arvA* strains, respectively, while it reduced metabolic activity by ~72% in the ∆*arvA* strain (Fig. 3B). These data suggest that loss of *arvA* confers a modest decrease in amphotericin B susceptibility in a submerged biofilm model. With regard to the triazoles, voriconazole was not effective against biofilms of the parental strain CEA10, as previously observed (2, 31). In this assay, voriconazole only reduced the metabolic activity by ~7% and 12% of CEA10 and the reconstituted strain ∆*arvA + arvA,* respectively (Fig. 3C). Voriconazole also did not reduce metabolic activity of the ∆*arvA* strain in this biofilm model (Fig. 3C). This is in contrast to what we observed in our agar-based colony biofilm assays (Fig. 2C). Taken together, these data suggest potential perturbations in the cell wall and/or lipid composition of the ∆*arvA* that impact antifungal drug susceptibility under specific conditions. Moreover, they highlight the differential impact of a given gene’s role in response to antifungals in different testing models (agar plate vs submerged biofilms).

With regard to the echinocandins, treatment with the β-glucan synthesis inhibitor caspofungin was highly efficacious against the parental and reconstituted strains at the time points analyzed. Caspofungin (0.125 µg/mL) reduced metabolic activity by 93% and 94% in CEA10 and ∆*arvA + arvA,* compared to only ~26% reduction in the ∆*arvA* strain. Treatment with a higher dose of caspofungin showed a similar trend, with a reduction of ~96% of metabolic activity in CEA10 and ∆*arvA + arvA*, compared to only ~16% in ∆*arvA*. This result confirms what we observed in agar-based colony biofilm plate assays (Fig. 3D) and supports that ∆*arvA* is less susceptible to β-glucan synthesis inhibition by caspofungin. Loss of *arvA* also increases susceptibility to calcofluor white. A low dose of CFW reduced ~60% and ~66% metabolic activity in CEA10 and ∆*arvA + arvA* (Fig. 3E). Strikingly, the same dose of CFW reduced ~96% of metabolic activity in ∆*arvA* biofilms (Fig. 3E). This increased susceptibility to CFW in ∆*arvA* was replicated with 25 µg/mL of CFW treatment (~97% reduction) (Fig. 3E). The *arvA*-dependent susceptibility to CFW replicated what we observed in agar-based colony biofilm assays (Fig. 2A and B), further supporting that loss of ArvA impacts the composition and/or function of the fungal cell wall.

### Loss of *arvA* induces changes in cell wall polysaccharide exposure

To test whether ∆*arvA* has changes in cell wall polysaccharide exposure, we imaged and measured chitin and β-glucan cell surface exposure by staining with wheat germ agglutinin (WGA) and soluble Dectin-1, respectively (32, 33). We grew biofilms (1 × 10^5^ conidia/mL) in liquid GMM for 24 hours prior to staining with FITC-WGA (5 µL/mL) and fixing with 4% paraformaldehyde. Biofilms stained with soluble Dectin-1-Fc (sDectin-1) were fixed prior to staining as indicated in Materials and Methods. Each strain with either WGA or sDectin-1 staining was imaged, and images were processed and normalized using FIJI software. Due to the morphological differences of ∆*arvA* (hyper-swollen conidia), we measured chitin and β-glucans of hyphae for appropriate comparison with the parental and reconstituted strain.

**Fig 4:**
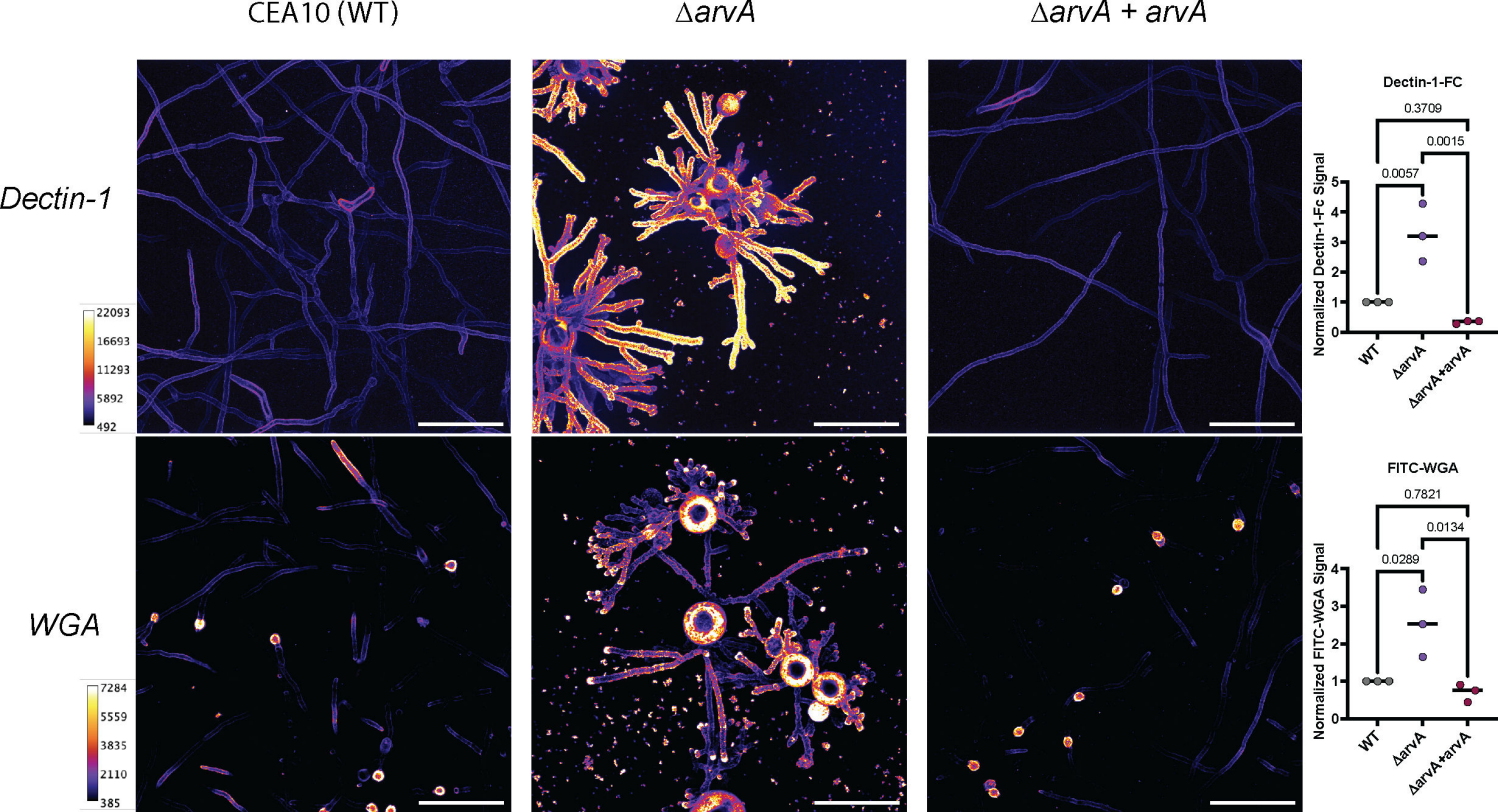
Loss of *arvA* shows increased exposure of chitin and β-glucans. A total of 1 × 10^5^ conidia/mL of CEA10, ∆*arvA,* and ∆*arvA + arvA* strains were inoculated into filtered liquid GMM in 8-well Ibidi plates and incubated for 24 hours at 37°C with 5% CO_2_. For WGA staining, biofilms were stained with 5 µL/mL of FITC-WGA, incubated at room temperature for 30 minutes, and subsequently washed with PBS. After washing, biofilms were fixed with 4% paraformaldehyde for 15 minutes. Soluble Dectin-1 staining was achieved by fixing biofilms with 4% paraformaldehyde for 15 minutes and staining with 5 µg/mL of sDectin-1-Fc before incubating with Alexa Fluor 488 anti-human IgG (ThermoFisher). Biofilms were imaged, and levels of exposed chitin and β-glucan were measured as described in Materials and Methods. Quantification data are representative of three biological replicates. Each biological replicate (*n* = 3) consists of measurements on 3 different fields of view, for a total of 12 independent measurements of mean gray value per biological replicate. Scale bars are 50 µm. Statistical significance was determined using an ordinary one-way ANOVA with Tukey’s multiple comparisons test. Heatmap indicates intensity values of images.

Both WGA and sDectin-1 were able to stain all three strains (CEA10*,* ∆*arvA,* and ∆*arvA + arvA*) (Fig. 4). Interestingly, the ∆*arvA* strain exhibited both WGA and sDectin-1 signals (fluorescent aggregates) in the medium, suggesting potential cell wall shedding (Fig. 4). This phenotype may suggest that the ∆*arvA* cell wall is more fragile than the WT and reconstituted strain and is more susceptible to mechanical disruption in the staining and fixing process. As expected, CEA10 and ∆*arvA + arvA* showed no statistical difference in either WGA or sDectin-1 (Fig. 4). WGA staining in CEA10 and ∆*arvA + arvA* strains was more prominent in the hyphal tips and in the conidia, while the ∆*arvA* strain showed a more equivalent staining throughout the entire fungal mass (Fig. 4). Additionally, WGA staining in ∆*arvA* is heavily punctate throughout the hyphae rather than the smooth, well-defined pattern observed on CEA10 and ∆*arvA + arvA* strains or even in sDectin-1 staining in ∆*arvA* (Fig. 4). This result suggests that ∆*arvA* has altered chitin localization in the hyphal cell wall. Overall, the ∆*arvA* strain had higher staining of both sDectin-1 and WGA compared to the WT and the reconstituted strains (Fig. 4). Together, these results suggest that the cell wall organization in ∆*arvA* hyphae differs from that of the wild-type and reconstituted strains.

### ∆*arvA* strain is fully virulent and can grow *in vivo* in mouse models of IPA

**Fig 5:**
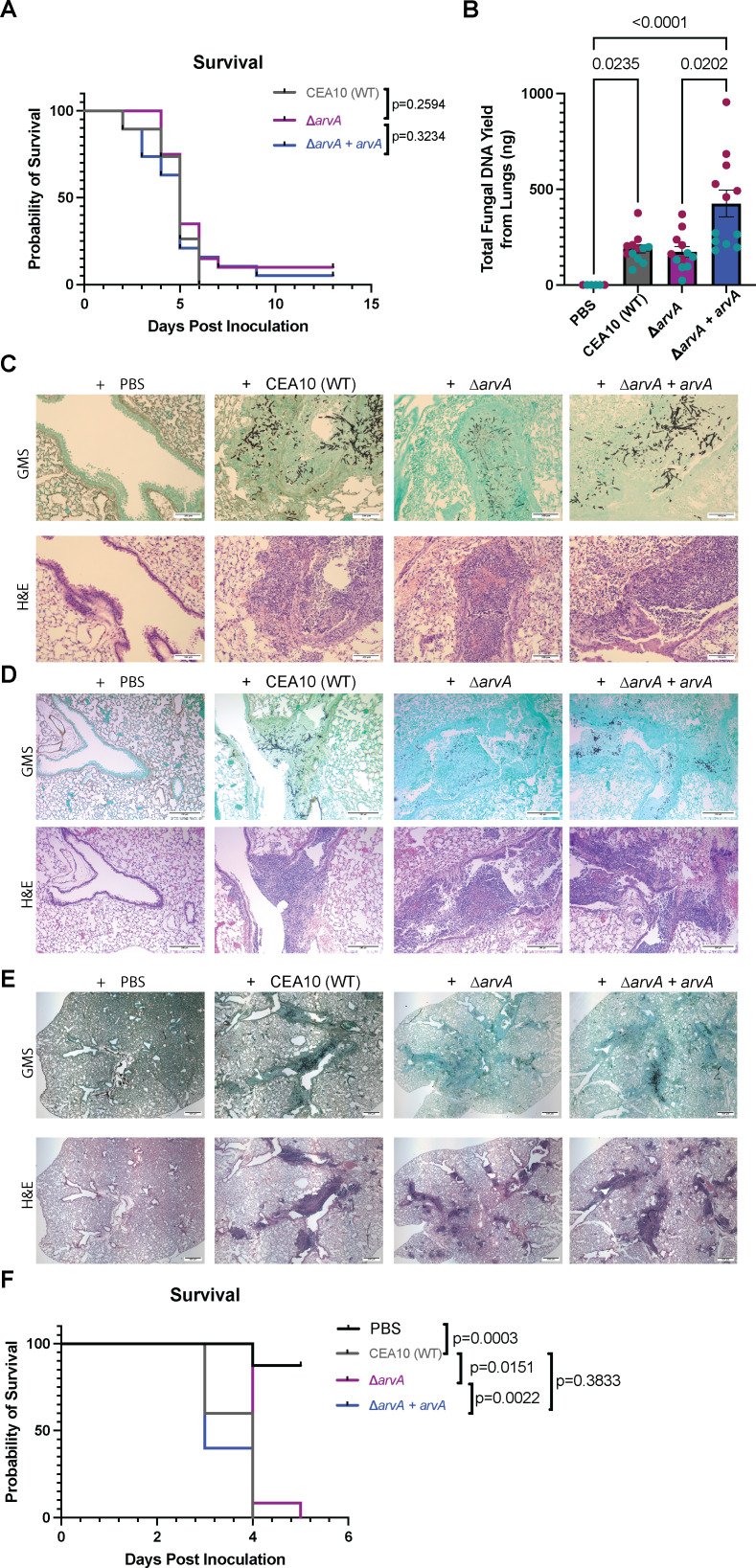
∆*arvA* is fully virulent and able to grow in the lungs but shows reduced tissue invasion in mouse models of IPA. (**A**) ∆*arvA* has no significant difference in murine survival relative to wild-type and reconstituted strains in an immunosuppressed steroid mouse model of invasive pulmonary aspergillosis. Outbred female CD-1 mice (22–24 g) (Charles River Laboratories) were injected subcutaneously with 40 mg Kenalog-10/kg of body weight (triamcinolone acetonide; Bristol-Myers Squibb, Princeton, NJ, USA). Twenty-four hours after steroid injection, mice were inoculated intranasally with 1 × 10^5^ conidia (WT-CEA10, ∆*arvA,* or ∆*arvA + arvA*) in 40 µL sterile PBS while under isoflurane anesthesia. Data are represented on a Kaplan-Meier plot to determine significance (18–20 mice/group from two independent experiments). (**B**) ∆*arvA* fungal burden is similar to wild type in murine lungs. Mice were injected with Kenalog-10 and inoculated as described above. At 3 days post-inoculation, select mice were euthanized, lungs were excised from the chest cavity, and genomic DNA was extracted for 18S rDNA qPCR quantification of fungal DNA. A one-way ANOVA with a Kruskal-Wallis test comparing all groups was used to determine significance (12 mice per group from two independent experiments). (**C**) ∆*arvA* shows reduced tissue invasion compared to wild-type and reconstituted strains. Mice were injected with Kenalog-10 and inoculated as described above. At 3 days post-inoculation, four mice per strain were euthanized, cannulated, and lungs were inflated with 10% buffered formalin phosphate. Lungs were paraffin-embedded and stained for hematoxylin and eosin and Gomori methenamine silver. H&E- and GMS-stained lungs were imaged at 20× objective using a standard upright light microscope fitted with an AmScope MU1000 camera. Scale bars were added using an AmScope calibration slide and ImageJ software (ImageJ2, version 2.14.0/1.54f). Scale bars are 200 μm. Data are representative of six to eight mice per group from two independent experiments. (**D**) Experimental details as in panel** C** except images taken at 10× and scale bar = 200 µm and (**E**) at 2.5× and scale bar = 500 µm. (**F**) ∆*arvA* is fully virulent in a leukopenic murine model of IPA. Six- to seven-week-old female CD-1 mice were injected intraperitoneally with 150 mg cyclophosphamide/kg of body weight on day −2 and subcutaneously with 40 mg Kenalog-10/kg of body weight on day −1. Twenty-four hours later, they were inoculated intranasally with 1 × 10^5^ conidia/40 µL PBS of the indicated strains. Survival was monitored over the course of 5 days. A Kaplan-Meier survival analysis and log-rank test comparing WT CEA10 to the other three groups were used to determine significance between isogenic sets. For PBS, *n* = 8; for WT and reconstituted strains, *n* = 10; and for ∆*arvA*, *n* = 12.

Given the significant *in vitro* growth and morphological defects of the ∆*arvA* strain, we hypothesized that loss of ArvA would inhibit *A. fumigatus* pathogenesis and/or significantly reduce virulence in murine models of invasive pulmonary aspergillosis. To test whether *arvA* is necessary for pathogenicity and virulence, we challenged Triamcinolone (steroid) immune-suppressed mice with the *arvA* isogenic strain set as previously reported (34, 35). On day 3 post-fungal challenge (dpi 3), we sacrificed mice to evaluate histology (Gomori methenamine silver [GMS] and hematoxylin and eosin [H&E]) and quantify lung fungal burden. In separate experiments, pathogenicity and virulence were quantified utilizing a Kaplan-Meier survival curve analysis (Fig. 5).

Surprisingly, the ∆*arvA* strain showed no significant difference in survival in this steroid-mediated IPA murine model compared to CEA10 and ∆*arvA + arvA* strains (Fig. 5A). Furthermore, ∆*arvA* and CEA10 had no significant difference in fungal burden as measured by qPCR from mouse lungs at 3 dpi (Fig. 5B). Given the likely increase in nuclei observed *in vitro,* we wondered if the qPCR assay might predict higher *arvA* null mutant strain fungal burden than is actually present in the lung. Histopathology examination suggests that the ∆*arvA* strain grows in the mouse lung, though it appears to be less robust than the parental and reconstituted strains (Fig. 5C and D). However, in spite of its stunted morphology *in vitro*, the ∆*arvA* strain exhibits no quantifiable evidence of a morphological difference *in vivo* compared to CEA10 and the reconstituted strain ∆*arvA + arvA* (Fig. 5C and D). Interestingly, the histopathology of the ∆*arvA* strain also suggests that the strain is largely contained within the larger airways and shows an apparent reduction in invasion into surrounding parenchyma, with some evidence of accumulation in the alveolar spaces (Fig. 5C and D). In contrast, CEA10 and ∆*arvA + arvA* strains are able to invade the lung parenchyma and proliferate (Fig. 5C and D). Further assessment of the histology slides at 2.5× magnification confirmed that the ∆*arvA* strain is largely restricted to the larger bronchioles with a modest presence in the alveolar spaces (Fig. 5E).

Consequently, histopathology suggests that mortality mechanisms may be different in this IPA model with ∆*arvA* compared to the parental and reconstituted strains. One possibility is that the ∆*arvA* strain induces hyperinflammation due to the changes in cell wall morphology and potential shedding of cell wall material, observed *in vitro*. Increased exposure of *A. fumigatus* β1,3-glucans is correlated with higher inflammation as a result of increased immune response *in vivo* (36, 37). To test whether the mortality observed in ∆*arvA*-challenged steroid-treated mice is due in part to immunopathogenesis, we conducted a pathogenesis experiment using a chemotherapeutic leukopenic murine model of IPA (Fig. 5F). Interestingly, similar to the steroid murine model, the *arvA* strain was fully virulent, although mortality was delayed by 1 day compared to WT and ∆*arvA* + arvA-infected mice. The mortality delay was statistically significant; however, as all ∆*arvA*-challenged mice succumbed to infection, its relevance for therapeutic development is likely low. We conclude that immunopathogenesis differences at least partially contribute to ∆*arvA*-induced pathogenicity in the steroid model, though other factors are likely in play to fully explain host mortality. Taken together, these data surprisingly suggest that loss of *arvA* does not substantially impact disease progression and mortality in two immunologically distinct murine models of IPA and raise the question of why the severe *in vitro* growth phenotype is not recapitulated *in vivo*.

### *In vitro A. fumigatus* growth requirements in the absence of *arvA*

**Fig 6:**
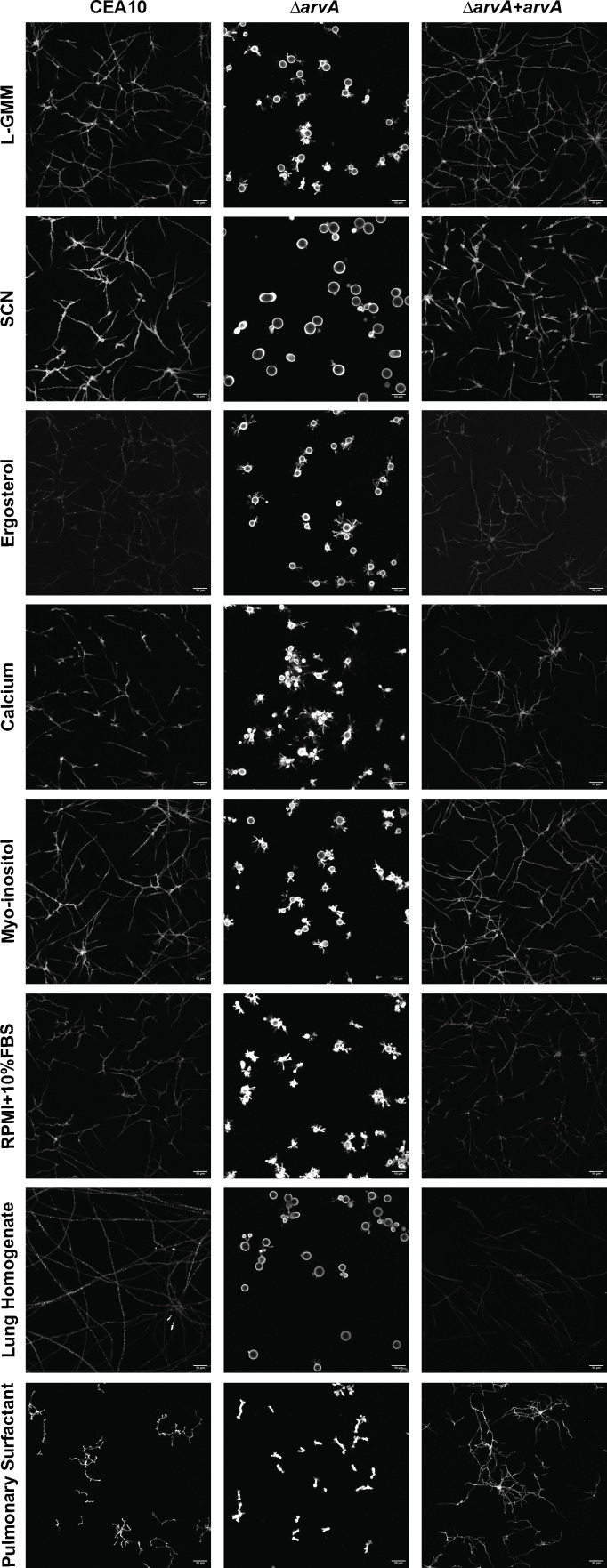
∆*arvA* is partially rescued in natural calf pulmonary surfactant. CEA10, ∆*arvA,* or ∆*arvA + arvA* were seeded at 1 × 10^5^/mL in L-GMM, SCN, RPMI + 10% FBS, lung homogenate, lung surfactant, or natural calf pulmonary surfactant media or L-GMM supplemented with calcium, myo-inositol, or ergosterol for 18 hours. Biofilms were stained with calcofluor white (10 µg/mL) and imaged on a confocal microscope at 60×, scale bars = 50 µm. Data are representative of three biological experiments with two technical replicates each.

We subsequently hypothesized that the ill-defined murine lung environmental conditions at least partially rescued the ∆*arvA in vitro* stunted growth and morphology. To try and identify what lung nutrient/condition might rescue ∆*arvA* morphology *in vivo*, we tested an array of different media (GMM, SCN, RPMI, and lung homogenate) and supplements (ergosterol, calcium, and myo-inositol). None of these conditions were able to rescue the ∆*arvA* growth and morphology we observed *in vitro* in GMM (Fig. 6). As we observed ∆*arvA* in some alveolar spaces in histopathology, we next tested growth between CEA10, ∆*arvA,* and ∆*arvA + arvA* in a natural calf pulmonary surfactant (used clinically) containing medium. As expected, culturing in surfactant reduced overall growth of CEA10 and ∆*arvA + arvA* strains compared to their growth in the relatively nutrient-rich liquid-glucose minimal media (L-GMM), while the ∆*arvA* strain had modestly improved germination compared to L-GMM (Fig. 6). Furthermore, ∆*arvA* morphology in pulmonary surfactant looked more similar to CEA10 and ∆*arvA + arvA* than in L-GMM (Fig. 6). While these data do not rule out other unknown host nutrients/conditions that promote pathogenicity and virulence in the absence of ArvA, they suggest that growth in a host relevant nutrient source, surfactant, is a better indicator of ∆*arvA* murine model outcomes.

## DISCUSSION

ArvA is conserved across eukaryotes and is important for proper lipid distribution in many organisms, though its precise molecular function remains unclear. In *A. fumigatus*, single gene replacement of the *arvA* gene resulted in stunted hyphal growth and visible changes in biofilm morphology in solid agar and liquid media assays. A hallmark of this stunted biofilm morphology is hyper-swollen, multinucleate conidia that continue swelling despite hyphal growth occurring (Fig. 1). It is unclear why the ∆*arvA* mutant conidia hyperswell, characteristic of isotropic growth, but simultaneously initiate multiple polarity axes as evidenced by the multiple germ tubes that arise from each conidium. This is highly atypical for *A. fumigatus*, as conidia usually swell from ~2 to ~5 µm and stop swelling after polarity is established and the first germ tube emerges (38, 39). These data strongly suggest that ArvA mediates both polarity establishment and maintenance in this filamentous fungus.

The mechanism and reason behind the altered germination progression are unclear but could be related to ArvA’s predicted function as an important lipid transporter associated with the endoplasmic reticulum. Membrane lipid organization and composition are important for the establishment and maintenance of polarized growth in other fungi, including *Aspergillus nidulans* and the dimorphic fungal pathogen *Candida albicans* (40). In *A. nidulans,* ceramide synthase mutants result in altered polarized growth morphologies, and loss of the lipid transporter encoding gene *dnfA* results in altered hyphal and colony morphology (41, 42). In *C. albicans*, steep gradients of phospholipids facilitate polarized growth in both the budding yeast and filamentous form (43, 44). Additionally, lipid transporters, specifically Dsr2, are important for invasive growth and virulence (45). Taken together, these studies support the hypothesis that *A. fumigatus* ArvA is a potential lipid transporter involved in organizing lipids important for key fungal life cycle morphological transitions. Future studies may employ lipidomics and/or membrane sterol profiling to assess the impact of ArvA loss on membrane composition during polarity establishment and maintenance.

Along these lines, multiple Hoescht-stained puncta in the *A. fumigatus arvA* null mutant suggest potential cell cycle progression dysfunction in the absence of *arvA*. Multinucleate cells, as a result of loss of function of *arvA,* have not been reported in other fungi (5, 6, 12, 13). This phenotype, however, has been reported in human cells with reduced levels of the homolog Arv1 (8). In these cells, Arv1 plays a role in cell cycle progression by recruiting IQGAP1 to the cleavage furrow (8). IQGAP1 is a plasma membrane-associated protein that regulates the actin cytoskeleton and is needed for cell polarity (46). In the fungal pathogen *C. albicans*, downregulating its IQGAP1 homolog, IQG1, results in multiple polarity sites that cause multi-budding (47). As the *A. fumigatus* ∆*arvA* strain has hyper-swollen conidia with multiple Hoechst-stained puncta and multiple germ tubes emerging, we hypothesize that ArvA plays a role in cell cycle progression through interaction with IQGAP1 homologs in *A. fumigatus,* similar to Arv1 in humans. This is an exciting and novel phenotype in fungi that could expand knowledge of cell cycle progression in *A. fumigatus*. Interestingly, Arv1 has been shown to translocate from the cleavage furrow to the intercellular bridge during the telophase stage of the cell cycle in human cells (8). Studying cellular localization of ArvA in *A. fumigatus* and potential interactions with the IQGAP1 homolog (Rho-family GTPase, AFUB_020720) is worthy of future investigations.

Further support of the importance of ArvA in *A. fumigatus* biology is evident by the altered antifungal drug susceptibility of the *arvA* null mutant. While the ∆*arvA* strain had a non-significant decrease in susceptibility to amphotericin B in the agar-based colony biofilm assays (Fig. 2A and B), in the XTT biofilm assay, ∆*arvA* had a surprisingly significant decrease in amphotericin B susceptibility (Fig. 3B). This result is surprising due to previous reports in *S. cerevisiae*, where a mutant *arv1* strain without the conserved AHD domain is hypersusceptible to amphotericin B (12). However, the assays and mutants in our study and the *S. cerevisiae* study are different, potentially explaining the difference in susceptibility observed. Regardless, the data indicate that loss of ArvA likely alters ergosterol/lipid membrane content, and thus assessing ergosterol and lipids in the cell membrane of ∆*arvA* biofilms in future studies is warranted.

With regard to triazoles, in the agar-based colony biofilm assay, ∆*arvA* in *A. fumigatus* has increased susceptibility to voriconazole (Fig. 2A and B), supporting previous reports of triazole susceptibility in ∆*arv1* strain in *C. albicans* and *S. cerevisiae* (6, 12, 26). Azole susceptibility in ∆*arv1* strains has been proposed to be due to Arv1p’s physical interaction with the azole target Erg11p, which stabilizes Erg11p and increases its half-life. Based on the azole susceptibility we observed with *A. fumigatus* ∆*arvA*, we hypothesize that ArvA likely plays a similar role in *A. fumigatus*. Additionally, we showed that loss of *arvA* likely results in altered cell wall composition and PAMP exposure. Relative to the parental strain (CEA10) and reconstituted strain (∆*arvA + arvA*), the ∆*arvA* strain has increased WGA staining, suggesting an increase in chitin exposure. Furthermore, ∆*arvA* is hypersusceptible to calcofluor white, which binds chitin in the cell wall. In other fungal species, Arv1 null mutants hyperaccumulate chitin and are hypersusceptible to calcofluor white (3, 20). As we did not measure chitin levels directly, we cannot conclusively say there is chitin hyperaccumulation in ∆*arvA* cell walls in *A. fumigatus*. However, based on the increased WGA signal (Fig. 4) and the conserved hypersusceptibility to calcofluor white (Fig. 2 and 3), which match observations in other fungal species, it is reasonable to speculate that there is increased chitin deposition in ∆*arvA*. Additionally, our microscopy showed irregular and punctate WGA staining in ∆*arvA*, suggesting that even though there is increased chitin in the cell wall, it is disorganized, which may lead to the increased calcofluor white susceptibility. This hypothesis is supported by the observation that increased cell wall chitin is associated with decreased susceptibility to echinocandins in *Candida* species (48), though it is unclear how alterations in the ∆*arvA* strain impact antifungal drug susceptibility, and it is worth considering whether these alterations impact drug penetration into the cell.

In yeast, ∆*arv1* has defects in β-glucan masking and increased levels of exposed β-glucans, even though the strain itself has reduced levels of both β-1,6- and β-1,3-glucans (49). In *A. fumigatus,* we observed increased binding of Dectin-1 to ∆*arvA* cells compared to the wild type (Fig. 4), consistent with a higher level of exposed β-glucans in the absence of *arvA*. Furthermore, ∆*arvA* had decreased susceptibility to caspofungin, which, based on the findings in yeast, we postulate is due to impaired synthesis of β-glucans. Alternatively, the likely increased chitin observed in the absence of ArvA may compensate for the loss of β-glucans. Interestingly, ∆*arvA* had cell wall fragments in the medium that stained with both WGA and Dectin-1, suggesting cell wall instability and shedding. Altogether, these results validate some of the published cell wall phenotypes of strains with loss of ArvA homologs in other fungi and introduce new questions regarding ArvA’s role in cell wall composition, assembly, and regulation of β-glucan synthesis in a filamentous fungus. As β-glucan synthase is localized to the cell membrane, it is possible that alterations in the membrane lipid content due to loss of ArvA lead to changes in β-glucan and chitin synthase activity and/or localization.

With significant growth and cell wall defects, it was expected that loss of ArvA would dramatically attenuate *A. fumigatus* pathogenicity and virulence. Consequently, perhaps the most surprising result of this study is the persistent pathogenicity and virulence of the ∆*arvA* strain in two immunologically distinct murine models of IPA (Fig. 5). The only observable differences in *in vivo* growth between ∆*arvA*, CEA10, and ∆*arvA + arvA* were a decrease in tissue invasion and perhaps a modest increase in ∆*arvA* presence in the alveolar spaces. Though limited by the resolution of histology imaging, to the extent that we could observe with this approach, the severe *in vitro* morphological and polarity defects of ∆*arvA* were absent *in vivo*. In the leukopenia model, disease progression is rapid and severe with all strains. It is possible that using lower conidial doses in this model would render larger differences between ∆*arvA*, CEA10, and ∆*arvA + arvA*-inoculated mice. We also considered whether conidial size differences may result in deposition differences but did not observe a significant difference in conidial cell size across strains. Consequently, we were curious if the nutrient environment of the airways rescued ∆*arvA* morphology and growth. However, differences in growth and morphology between the wild-type and ∆*arvA* strains persisted across numerous different *in vivo*-relevant nutrient sources that we examined *in vitro* (Fig. 6). Intriguingly, the one nutrient condition where we observed relatively similar growth and morphology among CEA10, ∆*arvA,* and ∆*arvA + arvA* was in natural calf lung surfactant. Importantly, surfactant did not fully rescue ∆*arvA* growth compared to CEA10 and ∆*arvA + arvA* growth in L-GMM; rather, it significantly restricted the growth of CEA10 and ∆*arvA + arvA* to better match ∆*arvA*. As this growth medium, which contains 35 mg/mL phospholipids (including phosphatidylcholine) and surfactant proteins B and C (0.7 mg/mL), is reflective particularly of the alveolar spaces in the lung, *in vivo* lung surfactant may be worth considering in the assessment of *A. fumigatus* mutants and strains as part of future studies. From this perspective, it is possible that ∆*arvA* is more resistant to surfactant-mediated fungal growth inhibition. For example, the surfactant protein D is known to restrict the growth of *A. fumigatus* (50). It would be of further interest to test if a co-culture experiment with primary differentiated epithelial cells would also rescue the ∆*arvA* morphology as seen *in vivo*. Taken together, these data raise the question of whether ArvA is a viable antifungal drug target for *A. fumigatus* due to the apparent *in vivo* complementation of ∆*arvA’s* severe growth defects. Future studies should examine infection outcomes with ∆*arvA* during antifungal drug treatment to determine if the increased efficacy of triazoles observed *in vitro* would occur in the *in vivo* infection microenvironment. Consequently, a significant take-home message of this study is that the still ill-defined pulmonary nutrient environment of an *A. fumigatus* infection makes predictions of pathogenicity and virulence, and thus the viability of a candidate gene product as a drug target, based on *in vitro* phenotypes, highly uncertain.

## MATERIALS AND METHODS

### Generation of the *arvA* mutant and the reconstituted gene

The *arvA* mutant was generated using a dual Cas9-gRNA system as previously described for *A. fumigatus* (51) (all primers used are listed in Table S1). Briefly, two crRNAs spanning the *arvA* gene were designed (g158, gacgaccgctctttgggcaa; g159, ggtcgaatgcgtcgtcccgc), which were separately annealed to tracrRNA to generate gRNAs. The gRNAs were conjugated with Cas9 to generate the Cas9 RNP complex. The repair template was generated from a hygromycin plasmid by PCR amplification using RAC 7051 and RAC 7052. Two micrograms of the repair template and the Cas9 RNP complex were transformed into *A. fumigatus* protoplasts as previously described (52).

To reconstitute the *arvA* null mutant, a plasmid was generated by amplifying the *arvA* gene locus using the primers RAC 7317 and RAC 7318. PCR using RAC 7319 and RAC 7320 primers and a ptrA-ampicillin vector was then utilized to generate a product with overhangs complementary to RAC 7318 and RAC 7317, respectively. Using HiFi assembly, a plasmid was generated from the two PCR products such that the *ptrA* selection marker is adjacent to the 3′ UTR of *arvA*. For transformation, the repair construct was amplified using primers RAC 7321 and RAC 6321, which carry flanks with 35–40 bp homology adjacent to a gRNA (g65, tctccttcataagcgaccag) cut site at the *Aft4* transposon (53). Transformation into the *arvA* mutant was performed as described above.

### Southern blot for verification of reconstituted strain copy number

A Southern blot was conducted as described previously (54). Briefly, 25 μg of WT, mutant, and the reconstituted strain gDNA was digested overnight using the *Hind*III enzyme and separated on a 1% agarose gel. The bands were transferred onto a positively charged nylon membrane and were hybridized overnight with a DIG-labeled probe specific to *ptrA*. Post-hybridization, washing, and DIG detection were carried out according to the manufacturer’s instructions (Roche).

### Agar-based colony biofilm drug susceptibility assays

A total of 1 × 10^3^ conidia in 2 μL of WT-CEA10, ∆*arvA* (CEA10), and ∆*arvA + arvA* (CEA10) strains were inoculated onto glucose minimal media plates [6 g/L NaNO_3_, 0.52 g/L KCl, 0.52 g/L MgSO_4_·7H_2_O, 1.52 g/L KH_2_PO_4_ monobasic, 2.2 mg/L ZnSO_4_·7H_2_O, 1.1 mg/L H_3_BO_3_, 0.5 mg/L MnCl_2_·4H_2_O, 0.5 mg/L FeSO_4_·7H_2_O, 0.16 mg/L CoCl_2_·5H_2_O, 0.16 mg/L CuSO_4_·5H_2_O, 0.11 mg/L (NH_4_)_6_Mo_7_O_24_·4H_2_O, 5 mg/L Na_4_EDTA, 1% glucose, and 1.5% agar; pH 6.5] treated with amphotericin B (0.75 µg/mL), voriconazole (0.0625 µg/mL), calcofluor white (25 µg/mL), or caspofungin (0.125 µg/mL). Plates were incubated at 37°C with 5% CO_2_ for 72 hours unless otherwise indicated. Colony diameter was measured using a ruler and normalized to percentage of the untreated control. Each experiment has three biological replicates with at least three technical replicates each.

### Biofilm drug assays

CEA10, ∆*arvA*, and ∆*arvA + arvA* strains were grown for 16 hours in liquid-glucose minimal media in either 96-well plates (XTT assay) or 6-well plates (biofilm biomass) at 1 × 10^5^ conidia/mL L-GMM in 5% CO_2_ at 37°C. ∆*arvA* conidia were grown in L-GMM for 34 hours under the same conditions as CEA10 and ∆*arvA + arvA* strains to match biomass. In 96-well plate cultures, the medium was removed and fresh L-GMM with either vehicle control, voriconazole (Sigma), caspofungin (Sigma), calcofluor white (VWR), or amphotericin B (Cayman Chemicals) was added for 3 hours. The medium was removed, and metabolic activity was assessed by addition of 0.5 µg/mL XTT (Thermo Scientific) plus 25 µM menadione (Enzo) for an additional hour, or until untreated control wells turned dark orange. Supernatants were transferred to a new plate, and optical density was measured at 450 nm. To assess the effect of drug treatment, metabolic activity was normalized to the percent reduction in metabolic activity relative to untreated control (biofilm damage) (2). Biofilm biomass was determined by collecting biomass from 6-well plates into a tube, washing two times with water, lyophilizing, and weighing.

### Dectin-1 and WGA staining

Dectin-1 and WGA staining were done according to reference 55. Briefly, 200 μL of 1 × 10^5^ conidia/mL in optically clear LGMM was added to an 8-well Ibidi slide at 37°C for 24 hours. For WGA staining, biofilms were stained with 5 µL/mL of FITC-WGA in optically clean LGMM, incubated at room temperature for 30 minutes, and subsequently washed with PBS. After washing, biofilms were fixed with 4% paraformaldehyde for 15 minutes. For biofilms stained with both WGA and CFW, CFW was added to fixed biofilms after WGA staining at a final concentration of 25 µg/mL.

Dectin-1 staining was achieved by fixing biofilms with 4% paraformaldehyde for 15 minutes. After fixation, biofilms were washed with PBS and then blocked for 20 minutes using RPMI supplemented with 10% fetal calf serum and 0.025% Tween 20. The blocking solution was removed, and 5 µg/mL of Dectin-1-Fc in blocking solution was added to the biofilms and was incubated at room temperature for 1 hour. Biofilms were washed with PBS, and a 1/300 dilution in PBS of Alexa Fluor 488 anti-human IgG (ThermoFisher) was added and incubated at room temperature for 1 hour. After this step, biofilms were washed with PBS and maintained in PBS until use.

### Microscopy

All samples were imaged on a Nikon spinning disk confocal microscope equipped with a Yokogawa CSU-W1 spinning head using a 40×, 60×, or 100× oil objective as indicated. To visualize FITC-WGA and Dectin-1 Fc/Alexa Fluor 488, a 488 nm laser was used. To visualize CFW, a 405 nm laser was used. Images were processed in Nikon Elements and Fiji software (56, 57). Images were acquired using z-stacks to capture the volume of the sample, and the same exposure time and laser intensity were used for all images within an experiment. For nuclear staining, 200 µL of 2.5e^5^ conidia per mL in liquid glucose minimal media was cultured in 8-well Ibidi slides at 37°C + 5% CO_2_ for 24 hours. After incubation, the medium was removed from the 24 hour biofilms, and they were fixed with 4% paraformaldehyde for 10 minutes at room temperature. Biofilms were washed and then permeabilized with 200 µL of 0.5% Triton X-100 in PBS for 10 minutes. After permeabilization, biofilms were washed with PBS and then stained with 200 µL of Hoescht 33342 nuclear stain (ThermoFisher) in PBS at a final concentration of 10 µM for 15 minutes at room temperature. After staining, biofilms were washed with PBS and maintained in PBS until imaging. Imaging was done on a Nikon Ti-E-inverted spinning disk microscope.

### Analysis of WGA and Dectin-1 fluorescent signal

To account for the different morphologies exhibited by ∆*arvA* in spore size, we quantified WGA and Dectin-1 on the hyphae. Images were analyzed using Fiji (56, 57). Using a line with a thickness of 10 in Fiji, we measured the intensity of the stained cell wall at a single z-slice, which is positioned in the center of the cell. We repeated this process in multiple hyphae per image. We then found the minimum (background) and maximum (peak) intensities and subtracted the average background intensities from the individual peak intensities per image.

### Microscopy to assess changes in ∆*arvA* morphology with different media

To perform screening for conditions that rescue cell morphology of the *arvA* mutant strain, 1 × 10^4^ conidia/mL were grown in the media indicated in either a standard 96-well plate for Fig. 6A, or 1 × 10^5^ conidia/mL inoculated into Ibidi glass-bottom slide dishes (Fig. 6B to D) (Cat. No: 80827) for 24 hours at 37°C with 5% CO_2_. For Fig. 6B to D, cells were stained with 25 μg/mL of calcofluor white approximately 30 minutes prior to imaging. Images were taken on a Nikon spinning disk confocal microscope using a 20× objective, using a 405 nm laser to visualize the CFW signal, except for in the ammonium condition, where DIC static images were taken due to interference with the CFW signal. Z-stacks were taken that encompassed the growth of the *arvA* mutant, and max projections of those Z-stacks are shown (raw images processed in FIJI). Images in panel A were taken using a camera attached to a standard benchtop light microscope using a 40× objective.

### Media preparation for ∆*arvA* morphology experiments

Glucose minimal media (as described in Agar-based colony biofilm drug susceptibility assays) was supplemented with 500 µg/mL myo-inositol or 25 mM CaCl_2_. Myo-inositol was resuspended in water, filter sterilized, and added to autoclaved GMM. For ergosterol supplementation, 2.5 mg of ergosterol was first resuspended in a 1 mL solution of 1:1 ethanol: Tween 80 and added at a 1:100 ratio to sterile GMM. SCN was prepared by adding 10 g/L glucose, 6 g/L NaNO_3_, 0.502 g/L KCl, 0.502g/L MgSO_4_∙7H_2_O, 1.52 g/L KH_2_PO_4_, 0.025 g/L MnCl_2_∙4H_2_O, 2.2 mg/L ZnSO_4_∙7H_2_O, 1.1 mg/L H_3_BO_3_, 0.5 mg/L MnCl_2_∙4H_2_O, 0.16 mg/L CoCl_2_∙5H_2_O, 0.16 mg/L CuSO_4_∙5H_2_O, 0.5 mg/L FeSO_4_∙7H_2_O, and 5 mg/L Na_4_EDTA; the pH was adjusted to 6.5 and autoclaved. After autoclaving, the following sterile solutions were added: L-glutamine solution to a final concentration of 146.14 mg/L, SC mix (Sunrise Science Catalog# 1300-030) to 1 g/L, and NaNO_3_ to 101.864 mg/L. RPMI 1640 with L-glutamine was purchased from Corning (product number 10-040-CV). RPMI + low glucose: 10.4 g/L of HyClone RPMI + 2.05 mM L-Glutamine Cat No: SH30011.04, D-glucose, 2 g/L. Fetal bovine serum was added to RPMI 1640 to a final concentration of 10% (FBS + RPMI). To generate lung homogenate medium, murine lungs were isolated, homogenized, and resuspended in 4 mL PBS. Natural calf surfactant (Infasurf; final concentration 100 µg/mL) was made by diluting in water with trace elements [2.2 g/L, ZnSO_4_·7H_2_O, 1.1 g/L H_3_BO_3_, 0.5 mg/L MnCl_2_·4H_2_O, 0.5 mg/L FeSO_4_·7H_2_O, 0.16 mg/L CoCl_2_·5H_2_O, 0.16 mg/L CuSO_4_·5H_2_O, 0.11 mg/L (NH_4_)6Mo_7_O_24_·4H_2_O, and 5 mg/L Na_4_EDTA].

### Microscopy to assess changes in ∆*arvA* morphology with pulmonary surfactant

Strains were seeded with 1 × 10^5^ conidia/mL for each indicated strain. Strains were grown in the indicated media in 24-well Ibidi plates for 24 hours at 37°C with 5% CO_2_. After growth, biofilms were stained with calcofluor white (10 µg/mL in PBS), and fluorescent images were taken on a Nikon Spinning Disc Confocal Microscope. Images were taken using a 405 nm laser.

### Murine model studies

Mice were randomly allocated into autoclaved cages at three to four mice per cage with HEPA-filtered air, with food and water available *ad libitum*. For the steroid model, outbred female CD-1 mice (22–24 g) (Charles River Laboratories) were injected subcutaneously with 40 mg Kenalog-10/kg of body weight (triamcinolone acetonide; Bristol-Myers Squibb, Princeton, NJ, USA). Twenty-four hours after Kenalog injection, mice were inoculated intranasally with 1 × 10^5^ conidia (CEA10, ∆*arvA,* or ∆*arvA + arvA*) in 40 µL sterile PBS while under isoflurane anesthesia. For the leukopenic model, mice were injected with 150 mg cyclophosphamide/kg of body weight intraperitoneally 48 hours prior to and 72 hours post-fungal inoculation. Mice were treated with Kenalog, inoculated, and monitored as described in the steroid model. For both models, mice were monitored three times per day for 14 days for signs of disease. Mortality data were analyzed with a Kaplan-Meier survival curve to determine significance between each group. See reference 58 for further details.

### Histopathology

CD-1 mice were housed, treated, and inoculated as described above. At 3 days post-inoculation, *n* = 4 mice/strain were euthanized, cannulated, and lungs were inflated with 10% neutral buffered formalin. Lungs were stored in 10% neutral buffered formalin for a minimum of 24 hours, then sectioned and stored in 70% ethanol until paraffin embedding. Paraffin-embedded lung sections were stained for H&E and GMS. H&E- and GMS-stained lungs were imaged at 40× objective using a standard upright light microscope fitted with an AmScope MU1000 camera. Scale bars were added using the AmScope calibration slide and ImageJ software (ImageJ2, version 2.14.0/1.54f).

### Fungal burden

CD-1 mice were housed, treated, and inoculated as described above. At 3 days post-inoculation, select mice were euthanized, and lungs were excised from the chest cavity, rinsed in sterile PBS, and flash frozen in liquid nitrogen. Lungs were then lyophilized and bead beaten with 2.3 mm zirconia beads. Genomic DNA was extracted according to the manufacturer’s protocol (E.Z.N.A. Fungal DNA Mini Kit; Omega Biotech), and modifications were made to the protocol as previously described (54). qPCR quantification of fungal DNA was performed as previously described (59).

## Data Availability

All underlying data are available in either the supplement or upon request from the authors.

## SUPPLEMENTAL MATERIAL

Table S1 msphere.00853-25-s0001.docx Primers.

ASM does not own the copyrights to Supplemental Material that may be linked to, or accessed through, an article. The authors have granted ASM a non-exclusive, world-wide license to publish the Supplemental Material files. Please contact the corresponding author directly for reuse.
