# Supplementary figures and images for: Insights into Aspergillus fumigatus morphogenesis and pathogenesis through the putative lipid transporter ArvA
Source: mSphere. 2026 May 15;11(6):e00853-25. doi: 10.1128/msphere.00853-25 (PMC13317216; doi:10.1128/msphere.00853-25)

**Supplementary Table 1:** List of all primers used in strain generation

**
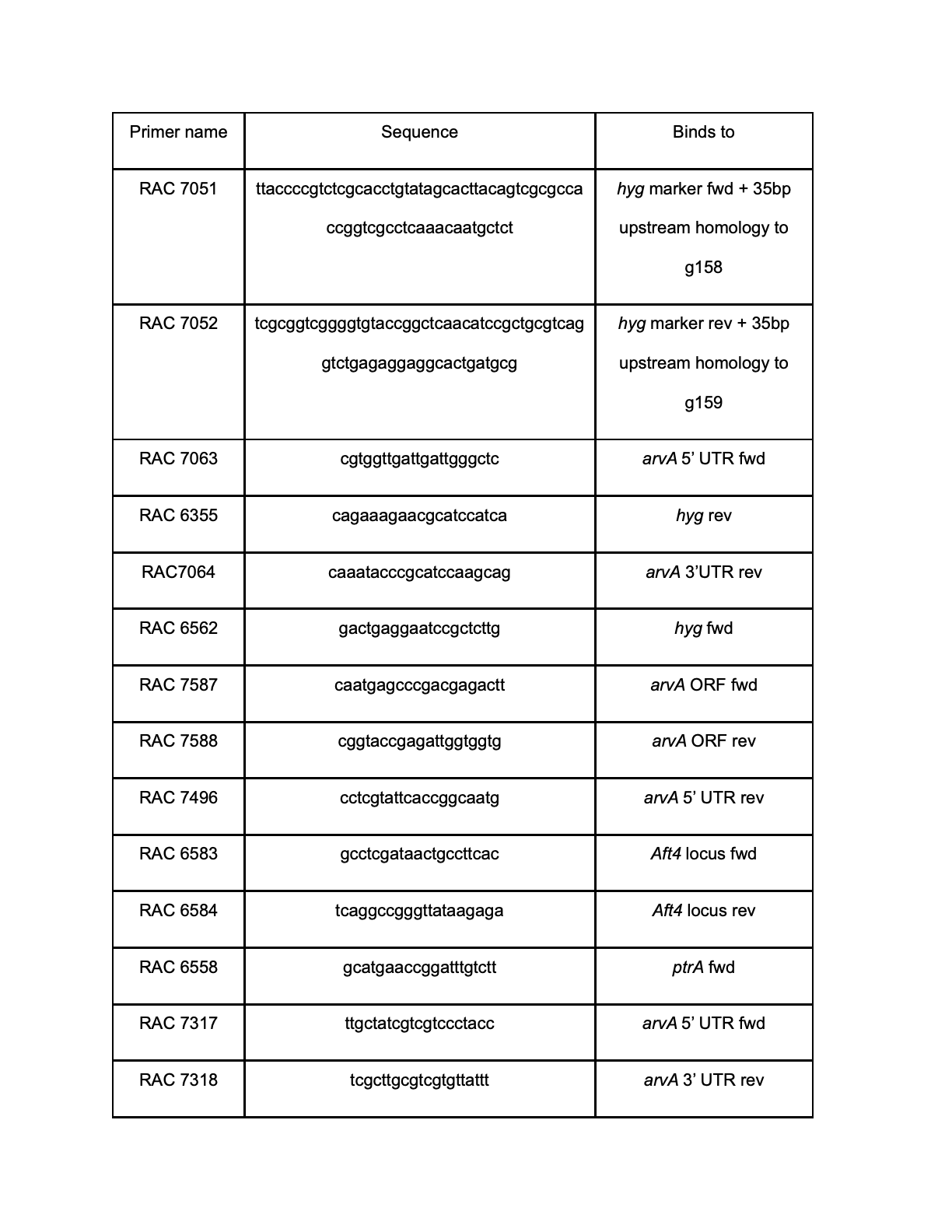
**

Supplement: Table S1 — Primers. [file msphere.00853-25-s0001.docx]
